# Fish Oil-Based Fat Emulsion Reduces Acute Kidney Injury and Inflammatory Response in Antibiotic-Treated Polymicrobial Septic Mice

**DOI:** 10.3390/nu8030165

**Published:** 2016-03-15

**Authors:** Juey-Ming Shih, Yao-Ming Shih, Man-Hui Pai, Yu-Chen Hou, Chiu-Li Yeh, Sung-Ling Yeh

**Affiliations:** 1 School of Nutrition and Health Sciences, College of Public Health and Nutrition, Taipei Medical University, Taipei 11031, Taiwan; shihcvs@gmail.com (J.-M.S.); yaomingshih2@gmail.com (Y.-M.S.); 2 Department of Surgery, Cathay General Hospital, Taipei 11073, Taiwan; 3 Department of Anatomy and Cell Biology, College of Medicine, Taipei Medical University, Taipei 11031, Taiwan; pai0507@tmu.edu.tw; 4 Master Program in Food Safety, College of Nutrition, Taipei Medical University, Taipei 11031, Taiwan; b006089078@tmu.edu.tw; 5 Department of Nutrition and Health Sciences, Chinese Culture University, Taipei 11114, Taiwan; m8707008@hotmail.com

**Keywords:** polymicrobial sepsis, acute kidney injury, *n*-3 PUFA, Treg cells, NGAL, inflammation

## Abstract

Acute kidney injury (AKI) is a common complication in sepsis. This study compared the effects of a fish oil-based with a mixed oil fat emulsion on remote renal injury in an antibiotic-treated septic murine model. Mice were randomly assigned to a normal control (NC) group and three septic groups. Sepsis was induced by cecal ligation and puncture (CLP). The antibiotic was injected intraperitoneally (IP) after CLP and then daily till the time of sacrifice. Three hours after antibiotic treatment, one of the septic groups was injected IP with a fish oil-based emulsion (FO), while the other two groups were given either a mixed oil emulsion (MO) or saline (SC). The septic groups were further divided into two separate time groups, with blood and kidneys samples collected at 24 h or 72 h post-CLP. The results showed that sepsis leads to the activation of neutrophils, T helper (Th)1/Th-2/Th-17 and Treg cells (*p* < 0.05). Plasma NGAL and mRNA expressions of renal MyD88 and TLR4 were also enhanced (*p* < 0.05). Compared to the SC group, the group given the fish oil-based emulsion had decreased plasma NGAL by 22% and Treg by 33%. Furthermore, renal gene expressions of MyD88 and TLR4 reduced by 46% and 62%, respectively, whereas heat shock protein 70 and peroxisome proliferator-activated receptor-γ increased by 158% and 69%, respectively (*p* < 0.05), at Day 3 after CLP. These results suggest that administration of a fish oil-based emulsion has favorable effects, maintaining blood T cell percentage, downregulating Treg expression, attenuating systemic and local inflammation and offering renal protection under conditions of antibiotic-treated polymicrobial sepsis.

## 1. Introduction

Acute kidney injury (AKI) affects approximately 35% of all ICU patients. This incidence correlates with an increase in in-hospital mortality from 20.9% to 56.8% depending on the severity of kidney injury [1]. Among the critically ill, sepsis is the leading cause of AKI [2]. Furthermore, septic patients have a greater risk of this developing into severe renal failure [3]. The pathogenesis of septic AKI is complex and multi-factorial. Traditionally, the host immune response in sepsis was considered to be initiated by a hyperinflammatory phase followed by a protracted immunosuppressive phase [4]. However, recent studies have shown that both pro- and anti-inflammatory responses occur early and simultaneously [5]. It is the imbalance between these two phases that results in a dysregulated inflammatory response, which may ultimately lead to irreversible multiorgan failure. A growing body of evidence suggests that the development of AKI under septic condition also involves the activation of both pro- and anti-inflammatory mechanisms. The concept that the principal mechanism leading to AKI in sepsis is more of a severely deregulated inflammatory response rather than a hemodynamic compromise (*i.e.*, ischemic AKI) is supported in part by clinical studies demonstrating pro-inflammatory cytokines (but not hypotension, nor vasopressor dosage) as a predictor of AKI among critically-ill patients [6]. This may explain why the presence of AKI often is an ominous sign in the syndrome of multiorgan failure.

Fish oils are rich sources of *n*-3 polyunsaturated fatty acids (PUFAs). An increase in the consumption of long-chain *n*-3 PUFAs, especially eicosapentaenoic acid (EPA, C20:5) and dosahexaenoic acid (DHA, C22:6), as found in oily fish and fish oil, has been shown to demonstrate many immunomodulatory and anti-inflammatory properties. The mechanisms elucidated thus far include competitive inhibition of arachidonic acid-derived inflammatory mediators, decreased activation of nuclear factor (NF)-κB and increased activation of peroxisome proliferator-activated receptor (PPAR)γ and other transcription factors [7]. Despite its anti-inflammatory effects, fish oil administration did not result in immunosuppression, as previously believed. In a rodent model of sepsis, Lin *et al.* [8] demonstrated that fish oil administration reversed the sepsis-induced reduction of the CD4 percentage and the CD4/CD8 ratio in splenocytes. Furthermore, Cao *et al.* [9] found that parenteral fish oil supplementation attenuated both the production of pro- and anti-inflammatory mediators, balanced the dysregulated inflammatory response and improved survival in septic rats. With regards to renal injury, *n*-3 PUFAs have been shown to possess some potential benefits on limiting the progression of chronic kidney disease in humans [10], as well as the preservation of renal function and improvement of survival in AKI induced by ischemia/reperfusion [11]. However, there is a paucity of current literature investigating the effects of fish oil supplementation on septic AKI, the most common and detrimental form of renal injury. In this study, we administered two commercial fat emulsions, a fish oil-based (Omegaven) and a mixed oil fat emulsion (SMOFlipid), to septic mice. Previous studies conducted on comparing these two fat emulsions focused their investigation mainly on liver disease. The studies found that Omegaven had more favorable effects on reducing hepatic steatosis and parenteral nutrition-associated liver disease than SMOFlipid [12,13]. We did not use an *n*-6 PUFA-rich fat emulsion, because it has been shown to exhibit proinflammatory and immunosuppressive effects and, therefore, is not recommended for use in critically-ill patients [14,15]. This study investigated the effects of Omegaven or SMOFlipid administered as a pharmaconutrient supplementation fed *ad libitum* after cecal ligation and puncture (CLP) on the development of AKI and associated inflammatory responses in a murine model of peritoneal sepsis. The histology and expressed inflammatory mediators of kidney were examined. Because dysregulated CD4 T cell subsets play an important role in the induction and continuance of sepsis [16], the alteration of CD4 T cell response was also assessed in this study. The CLP model was chosen to induce sepsis for its well-established similarity to bowel perforation with polymicrobial infection [17]. In order to establish a condition more closely mimicking the critical care setting, post-CLP animals were resuscitated by IV hydration and treated with an antibiotic.

## 2. Materials and Methods

### 2.1. Animals

Sixty-eight male ICR mice (6–8 weeks old, weighted 30–35 g) were obtained from the National Laboratory Animal Center (Taipei, Taiwan). Animals underwent an acclimatization period for 7 days before use in experiments. All mice were housed in a temperature- and humidity-controlled room and maintained on a 12-h light-dark cycle with food and water available *ad libitum*. Care of laboratory animals was in full compliance with the Guide for the Care and Use of Laboratory Animals (National Research Council, 1996). Experiment protocols were approved by the institutional Animal Care and Use Committee at Taipei Medical University (LAC-2013-0071).

### 2.2. Experimental Design

Mice were randomly assigned to a normal control group (NC, *n* = 8) and 3 septic groups at 2 time points (10 animals each at either 24 h or 72 h): a saline group (SC, *n* = 20), a fish oil group (FO, *n* = 20) or a mixed oil group (MO, *n* = 20). Sepsis was induced by CLP as previously described [18]. Animals were anesthetized with intraperitoneal (IP) injection of pentobarbital sodium (70 mg/kg). After midline laparotomy, the cecum was exteriorized and then ligated at the half-point from its end to the ileocecal junction using a 3-0 silk (Ethicon Inc., Somerville, NJ, USA) surgical tie. The cecum was then punctured “through and through” using a 23 G needle, resulting in 2 perforations. Fecal content (about 1 mm in diameter) was then squeezed from one perforation and then smeared onto the cecum. After the cecum was repositioned back into the peritoneal cavity, the wound was repaired in layers using the 3-0 silk continuous suturing technique. Immediately after the procedure, animals were resuscitated with 1 mL of 0.9% saline injected subcutaneously (0.5 mL behind the neck, 0.5 mL in the lower back). All CLP procedures were performed by a single operator. After surgery, animals were then returned to their cages. All animals were given standard rodent chow (Purina No. 5001) and allowed free access to food and water throughout the study. An antibiotic (75 mg ertapenem/kg, MERCK & CO., Inc., Whitehouse Station, NJ, USA) was given IP to all CLP animals starting 3 h after surgery and then daily over the course of the experiment. Three hours after antibiotic treatment, animals were randomized into 3 experimental groups (SC, FO, MO) to receive daily IP injection of 0.9% saline (SC), 10% (w/v) fish oil-based fat emulsion (FO, Omegaven, Fresenius-Kabi, Homburg, Germany) or 10% (w/v) mixed oil fat emulsion (MO, SMOF: contains 1.5% fish oil, 2.5% olive oil, 3% MCT, 3% soybean oil, Fresenius-Kabi, Homburg, Germany). In order to keep the amount of lipid supplement comparable between the two fat emulsion groups, the 20% SMOF was diluted to a 10% concentration with 0.9% saline in a ratio of 1:1, which was then administered at a dose of 10 mL/kg BW/day, the same as the fish oil-based emulsion. For the animals sacrificed at 24 h after CLP, a single dose of fat emulsion (Omegaven or SMOFlipid) supplement was administered 6 h after CLP. For those assigned to the 72 h study, 2 additional doses of fat emulsions were delivered on post-CLP Days 1 and 2, respectively, prior to the day of sacrifice.

The amount of fat used was equivalent to 1 g/kg/day. This dosage was lower than the parenteral dose used previously in rodents [19]. Since the energy expenditure of mice is about 3-times that in humans, this dose equates to nearly 0.3 g/kg/day of FO in humans [20], which is much higher than the optimal range for pharmaconutrient action, as described by Heller *et al.* [21]. In this study, the SC group was treated as a positive control group, since the additional fat emulsion administration in the intervention groups (FO, MO) provided only 1% more energy than the SC group. The septic groups received almost identical energy during the experimental period. Animals were euthanized by cardiac puncture at either 24 h or 72 h after CLP for sample collection and analysis, with 10 mice in total for each time group. The fatty acid compositions of the lipid emulsions are shown in Table 1.

Blood samples were collected in heparinized tubes for analysis of leukocyte population. Plasma was then separated from whole blood by centrifugation at 1500× *g* at 4 °C for 10 min. The peritoneum was opened and irrigated with 5 mL/100 g BW of saline. The plasma and peritoneal lavage fluid (PLF) were then stored at −80 °C for further analysis. Half of the right kidney was harvested and fixed with 4% paraformaldehyde in 0.1 M phosphate buffer for histological analysis. The remaining kidney tissues were snap frozen in liquid nitrogen and stored at −80 °C for further analysis.

### 2.3. Plasma Biochemical Markers and Inflammatory Mediators

A summary of the various variables measured is provided as followed: (1) systemic kidney function markers: blood urea nitrogen (BUN), creatinine (Cr), neutrophil gelatinase-associated lipocalin (NGAL); (2) biochemical markers: aspartate aminotransferase (AST) and alanine aminotransferase (ALT); and (3) inflammatory markers: prostaglandin (PG)E_2_ and lipoxin (LX)A_4_. BUN, Cr, AST and ALT were analyzed by the VetTest^®^ Chemistry Analyzer (IDEXX Laboratories Inc., Westbrook, MN, USA). NGAL (R&D Systems Inc., Minneapolis, MN, USA), PGE_2_ (R&D Systems Inc.) and LXA_4_ (MyBioSource, Inc., San Diego, CA, USA) concentrations were measured using a commercially-available enzyme-linked immunosorbent assay (ELISA) kit. Antibodies (Abs) specific to mice NGAL, PGE_2_ and LXA_4_ were coated onto the wells of the microtiter strips. The plates were incubated with plasma samples and developed with reagents. The absorbance of each well was measured using a spectrophotometer.

### 2.4. Blood Leukocyte Distribution

Flow cytometry was used to analyze the distribution of peripheral blood leukocytes and T cell subpopulation in whole blood. Each blood sample was divided into 100 μL in each aliquot, and these were then incubated with the different antibodies as described below. The antibodies used to detect leukocytes were as follows: PerCP-conjugated anti-CD45 (Biolegend, San Diego, CA, USA) for leukocytes, PE-conjugated anti-F4/80 (eBioscience, San Diego, CA, USA) for monocytes/macrophages and FITC-conjugated anti-Ly6G (BD Biosciences, San Jose, CA, USA) for neutrophils. For analysis of the T cell subpopulation, blood was incubated with PerCP-conjugated anti-CD45 (Biolegend), APC-conjugated anti-CD3ε (eBioscience), FITC-conjugated anti-CD4 (eBioscience) and Pacific blue-conjugated anti-CD8 (Biolegend) antibodies. The concentrations of antibodies were compliant with those recommended by the manufacturer. After 30 min of incubation at 4 °C in the dark, the lysed red blood cells were first suspended in staining buffer and then analyzed with a FACS Canto II flow cytometer (BD Biosciences). CD45-positive cells were gated for analysis, with the results presented as a percentage of specific CD-marker-expressing cells in blood leukocytes.

### 2.5. CD4^+^ T Cell Subsets in Blood

To determine the phenotypes of Th cells, 100 μL aliquots of blood were incubated with Pacific blue-conjugated anti-CD4 (BD Biosciences), fixed and then permeated for intracellular cytokine staining. The following Abs were used for intracellular cytokine staining: Alexa Fluor 488-conjugated anti-IL-4 (Biolegend), APC-conjugated anti-interferon (IFN)-γ (BD Biosciences) and PE-conjugated anti-IL-17A (Biolegend) Abs. For analyzing the Treg subset, leukocytes were incubated with Pacific blue-conjugated anti-CD4 and APC-conjugated anti-CD25 (eBioscience) Abs. After incubation for 30 min, leukocytes were fixed and permeated with Foxp3 staining buffer (eBioscience). PE-conjugated anti-Foxp3 (Biolegend) Abs were then added to stain for intracellular Foxp3. After RBCs were lysed, the leukocytes were stained and analyzed by flow cytometry. CD4-positive lymphocytes were gated on the basis of low forward and side scatter. Phenotypes of Th cells are presented as percentages of Th-associated cytokine-expressing cells in CD4-positive lymphocytes. The Treg population is presented as a percentage of CD25/Foxp3 double-positive cells in gated CD4-expressing lymphocytes.

### 2.6. Inflammatory Cytokine Concentrations in PLF

Local inflammatory markers, including IL-1β, tumor necrosis factor (TNF)-α, IL-6 and IL-10, were analyzed using ELISA in a microtiter plate. Antibodies specific to mice IL-1β, TNF-α, IL-6 and IL-10 were first coated onto the wells of the microtiter strips provided by the manufacturer (eBioscience, San Diego, CA, USA), incubated with the samples and then developed with reagents. The absorbance of each well was measured using a spectrophotometer.

### 2.7. RNA Extraction and Real-Time Polymerase Chain Reaction in Kidney

Total RNA of a 20 mg renal specimen of the kidney was obtained using TRIzol reagent (Invitrogen, Carlsbad, CA, USA). Complementary (c)DNA was synthesized from the RNA obtained using a RevertAid™ first-strand cDNA synthesis kit (Fermentas, Vilnius, Lithuania) according to standard protocols. Specific mRNA genes were amplified by real-time RT-PCR using the 7300 Real-Time PCR System (Applied Biosystems, Foster City, CA, USA). Primer sequences used for the quantitative RT-PCR assays are listed in Table 2 to include the following: (1) inflammatory-related genes: high mobility group box protein-1 (HMGB1), myeloid differentiation factor 88 (MyD88), toll-like receptor 4 (TLR4), TNF-α; and (2) anti-inflammatory genes: heat shock protein 70 (HSP70), peroxisome proliferator-activated receptor (PPAR-γ). Amplification was carried out in a total volume of 25 μL containing 1x Power SYBR Green PCR Master Mix (Applied Biosystems), 400 nM of each primer and 100 ng of cDNA. The reaction was carried out by starting with 1 cycle of 2 min at 50 °C and 10 min at 95 °C followed by 40 cycles of 15 s at 95 °C and 1 min at 60 °C, with the process verified by a final dissociation curve (DC) analysis. Cycle threshold (CT) values for each gene of interest were normalized to mice β-actin and expression levels of messenger (m)RNA were calculated by the equation 2^−ΔΔCT^ (ΔCT indicates the difference in threshold cycles between the test gene and β-actin, and ΔΔCT indicates the difference in ΔCT between the septic groups and sham group).

### 2.8. Renal Histology

Histological analysis of the kidneys was evaluated by a single pathologist who was blinded to the study groups. After organ removal, tissue was fixed by immersion in 4% buffered paraformaldehyde and embedded in paraffin. The paraffin-embedded kidney specimens were sliced into a thin five-micrometer thickness and stained with periodic acid-Schiff reagent (PAS) (Sigma Chemical, St. Louis, MO, USA). Standard hematoxylin (Sigma) nuclear staining was also applied to contrast the cell nuclei. PAS techniques were used to evaluate the polysaccharides, neutral mucosubstances, microvilli and basement membrane in tissues. Interpreting histological results to be favorable or unfavorable depends exclusively on the location of PAS accumulation. The presence of PAS-positive intensity in the apical border of the tubular epithelium indicates that the epithelium is intact, whereas excessive PAS expressions located in the basement membrane often suggests a thickening of the basement membrane, which may serve as a visual hint of an impaired renal function. Histological changes in the cortex and medulla were assessed collectively by quantitative measurements to reflect various degrees of kidney damage. The scoring system used to grade the degree of kidney injury was adopted from the criteria described by Kurus *et al.* [22], but with some modifications. Tubular damage was defined as the presence of tubular epithelial swelling, loss of brush borders, vacuolar degeneration, necrotic tubules, cast formation and desquamation. To assess the degree of kidney damage, at least 10 microscopic fields per specimen at 200× magnification were evaluated by a digital image analysis system (Image Pro Plus 5.1, Media Cybernetics, Silver Spring, MD, USA), with the degree of severity estimated after obtaining 3–6 independent samples from each group.

### 2.9. Statistical Analysis

All data are expressed as the mean ± the standard error of the mean (SEM). All analyses were conducted using GraphPad Prism 5 (GraphPad Software, La Jolla, CA, USA). The analysis of variance (ANOVA) with Tukey’s *post hoc* test was used to explore the differences among groups for all data, except for the analysis of histology. Histological scores were analyzed by the non-parametric Kruskal–Wallis test. A *p*-value of <0.05 was considered statistically significant.

## 3. Results

### 3.1. Survival Rates and Body Weights

All animals subjected to CLP survived till the time of sacrifice, either at 24 h or 72 h post-CLP. There were no significant changes in the body weights and visual physical activity of animals among the three CLP-induced septic groups during the experimental period.

### 3.2. Plasma Biochemical Parameters

In all of the septic groups at 24 h post-CLP, plasma levels of ALT and AST increased several folds as compared to the NC group regardless of the treatment. By 72 h, their elevated levels diminished somewhat, but still remained statistically higher than the NC group. Plasma BUN levels showed no significant increase after CLP and had no differences between all septic groups at both time points. In regards to serum creatinine, both the saline and the fat emulsion groups had higher Cr levels than the NC group by 24 h post-CLP; however, compared to the FO-3 and MO-3 groups, the Cr level of the SC-3 group continued to rise till the 72-h time point. Plasma NGAL in all septic groups increased dramatically by 24 h post-CLP. Although diminished levels were noted by 72 h post-CLP, their levels still remained significantly higher than that of the NC mice. However, when comparing among 72 h septic groups, the FO group (FO-3) had a significantly lower plasma NGAL level compared to the saline group (SC-3) at 72 h. (Table 3).

### 3.3. Blood Leukocyte Distribution

**Figure 1:**
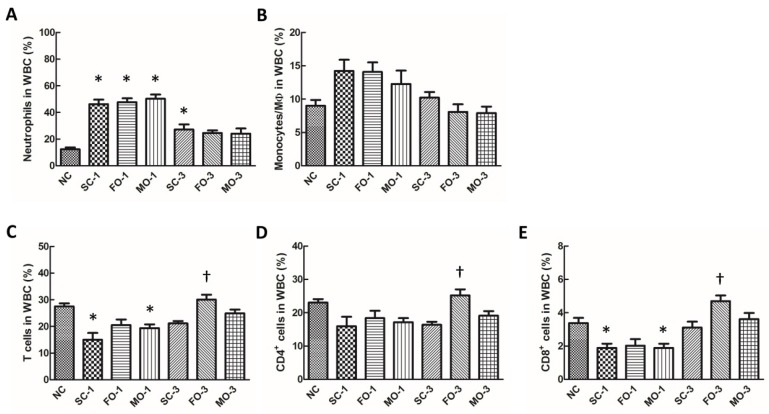
Proportion of (**A**) neutrophils; (**B**) monocytes and (**C**) T cells among leukocytes and percentages of (**D**) CD4+ and (**E**) CD8+ T cell subpopulations in blood. NC, normal control group; SC, septic group with saline injection; FO, septic group with fish oil (Omegaven) injection; MO, septic group with mix oil (SMOFlipid) injection. SC-1, FO-1, MO-1: sacrificed at one day after CLP; SC-3, FO-3, MO-3: sacrificed at three days after CLP. Values are presented as the mean ± SEM. N = 8 for NC, and *n* = 10 for the other experimental sepsis groups. Differences between groups were analyzed by ANOVA with Tukey’s post-hoc test. * Significantly different from the NC group (*p* < 0.05). ^†^ Significantly different from saline-treated groups at the same time points (*p* < 0.05).

By 24 h after CLP, the percentage of neutrophils was significantly increased in all septic groups, whereas the percentages of T and CD8+ cells were lower in the saline- and MO-treated mice, as compared to the NC group. No differences were observed in monocyte/macrophage percentages among the all groups (Figure 1A–C,E). When comparing the fat emulsion-treated groups to the saline group, only the FO-3 group demonstrated a higher percentage of T cells, CD4+ and CD8+ cells by 72 h post-CLP (Figure 1C–E).

**Figure 2:**
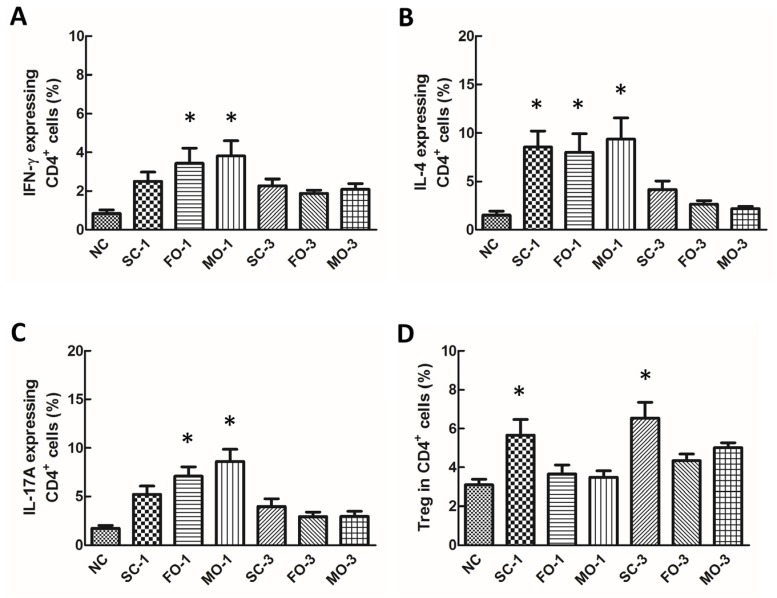
Percentage of Th1, Th2 and Th17 cell subpopulations and regulator T cells among CD4+ T cells in blood. Lymphocytes were gated according to their size and granularity using light scatter detectors (FSC/SSC). CD4-positive lymphocytes were considered Th cells and were gated to determine the expression of intracellular cytokines. Percentages of (**A**) interferon (IFN)-γ-, (**B**) interleukin (IL)-4- and (**C**) IL-17A-expressing CD4+ lymphocytes and (**D**) Treg cells. NC, normal control group; SC, septic group with saline injection; FO, septic group with fish oil (Omegaven) injection; MO, septic group with mix oil (SMOFlipid) injection. SC-1, FO-1, MO-1: sacrificed at one day after CLP; SC-3, FO-3, MO-3: sacrificed at three days after CLP. Data are presented as the mean ± SEM. N = 8 for NC, and *n* = 10 for other experimental sepsis groups. Differences between groups were analyzed by ANOVA with Tukey’s *post hoc* test. * Significantly different from the NC group (*p* < 0.05).

When assessing the different lineages of helper T cells after CLP, proportions of IFN-γ-expressing CD4+ cells (Th1 cells), IL-4-expressing CD4+ cells (Th2 cells) and IL-17A-expressing CD4+ cells (Th17 cells) all increased significantly in the fat emulsion-treated groups as compared to the NC group at 24 h (Figure 2A–C). The only significant increase of the helper T cell subset in the saline-treated group is the Th2 cell type (Figure 2B). Furthermore, a significant increase in the proportion of Tregs was only observed in the saline-treated groups (Figure 2D). In contrast, those of the NC group and the septic groups treated with fish oil and mixed oil emulsions showed no significant change in the Tregs proportion with levels similar to the NC group (Figure 2D).

### 3.4. Concentrations of Eicosanoids in Plasma and Cytokines in PLF

Levels of plasma PGE_2_ and LXA_4_ did not change significantly among the septic groups at both time points. At 24 h post-CLP, both IL-6 and IL-10 levels increased nearly two-fold in the FO group compared to the SC group. The MO group also exhibited a significant increase in plasma IL-6 level, but lacked the simultaneous increase of IL-10 level seen with the FO group. In addition, IL-1β and TNF-α levels showed significant elevation in the FO group by 72 h post-CLP, as compared to the SC group (Table 4).

### 3.5. Expression of Inflammation-Related Genes in Renal Tissues

**Figure 3:**
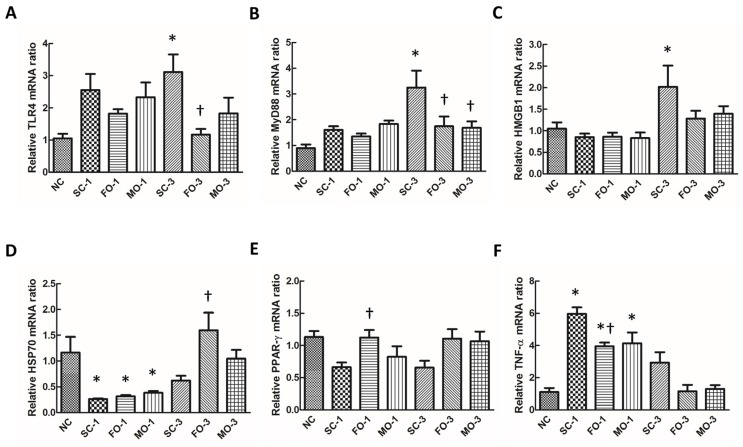
mRNA expressions of (**A**) toll-like receptor (TLR)4; (**B**) myeloid differentiation factor (MyD)88; (**C**) high-mobility group box protein (HMGB)1; (**D**) heat shock protein (HSP)70; (**E**) peroxisome proliferator-activated receptor (PPAR)-γ and (**F**) tumor necrosis factor (TNF)-α in kidneys at 24 h and 72 h after cecal ligation and puncture (CLP) measured by real-time RT-PCR. NC, normal control group; SC, septic group with saline injection; FO, septic group with fish oil (Omegaven) injection; MO, septic group with mix oil (SMOFlipid) injection. SC-1, FO-1, MO-1: sacrificed at one day after CLP; SC-3, FO-3, MO-3: sacrificed at three days after CLP. Values are presented as the mean ± SEM. N = 6 for each group. Differences between groups were analyzed by ANOVA with Tukey’s *post hoc* test. * Significantly different from the NC group (*p* < 0.05). ^†^ Significantly different from saline-treated groups at the same time points (*p* < 0.05).

Septic groups had higher TNF-α gene expression than the NC group at 24 h after CLP. Furthermore, the expressions of MyD88, TLR4 and HMGB-1 genes were also higher in the SC-3 group compared to the NC group. There were no difference in HMGB-1 expression between the NC, FO and MO groups. Both fat emulsion-treated groups had significantly lower MyD88 mRNA expressions when compared to the saline group at 72 h post-CLP (Figure 3A–C,F); furthermore, the fish oil-treated group had the least increase in TNF-α and TLR4 gene expressions at 24 h and 72 h, respectively, in which the level was significantly lower when compared to the saline-treated group (Figure 3A,F). PPAR-γ and HSP70 mRNA expressions in renal tissue were significantly higher in the fish oil-treated mice, as compared to the saline group at 24 h and 72 h after CLP, respectively (Figure 3D,E).

### 3.6. Histological Findings of Renal Tissue

**Figure 4:**
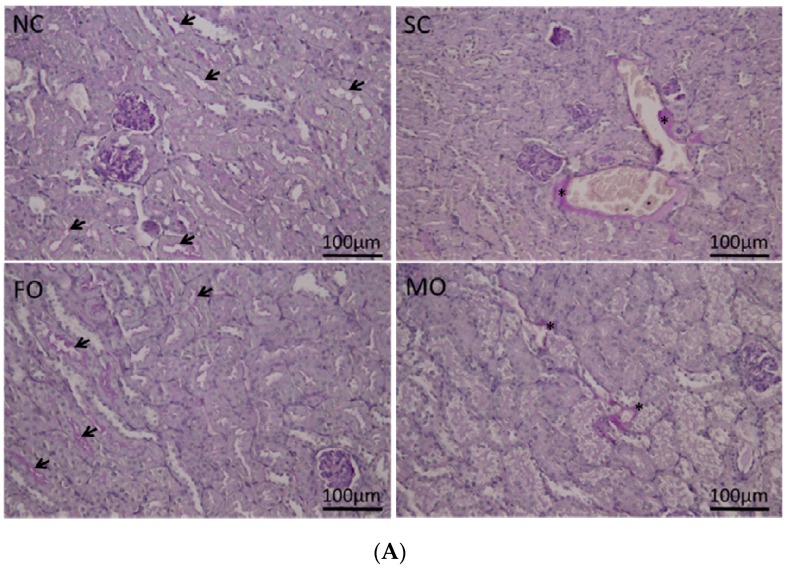

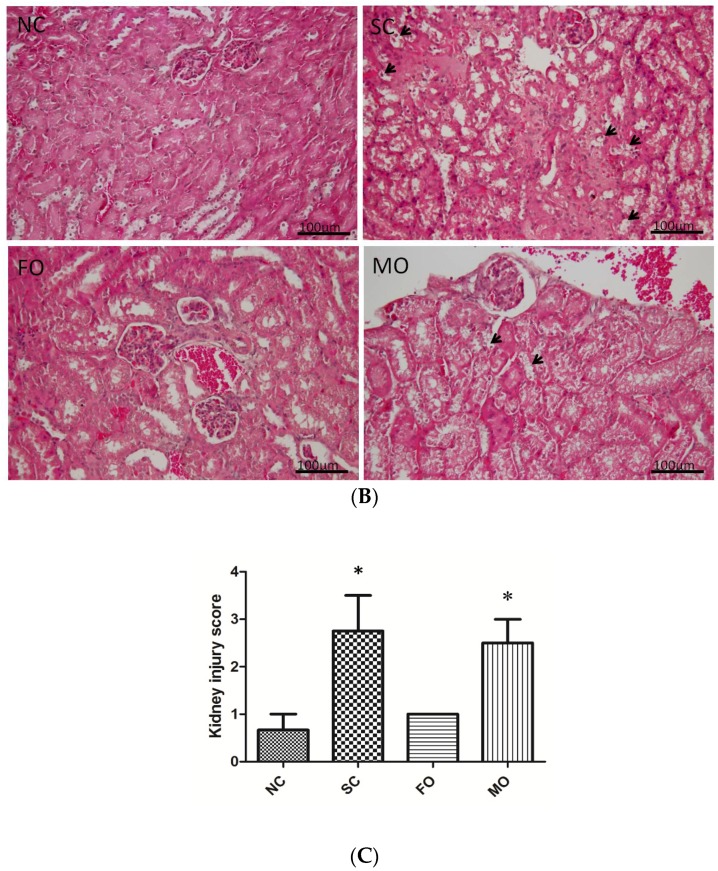
Histology and quantification of kidney tissues at 72 h after CLP. (**A**) NC and FO groups showed pale pink, periodic acid-Schiff reagent (PAS)-positive intensity in the apical border (arrow heads) of the tubular epithelium. Scattered PAS expressions (asterisk) were seen between the renal tubules in the SC and MO groups; (**B**) Hematoxylin and eosin staining of the NC and FO groups showed normal renal tubules without inflammatory changes. Dilated renal tubules, swollen tubular cells (arrow heads) and vacuolar degeneration were seen in the SC and MO groups; (**C**) Quantification of histological renal injury scores. NC, normal control group; SC, septic group with saline injection; FO, septic group with fish oil (Omegaven) injection; MO, septic group with mix oil (SMOFlipid) injection. The results are expressed as the mean ± SEM; *n* = 6 for each group. Differences between groups were analyzed by the non-parametric Kruskal–Wallis test. * Significantly different from the NC and the FO groups (*p* < 0.05).

NC and FO groups showed pale pink, PAS-positive intensity in the apical border of the tubular epithelium, indicating that the epithelium is intact. The presence of some patchy PAS expressions seen in the basement membrane of renal tubules in the SC and MO groups (Figure 4A) suggested scattered thickening of the basement membrane, which may be interpreted as visual evidence of impaired renal function. There was cast formation in the SC group. Consistent with PAS expression, histological findings of H&E staining showed normal renal tubules without inflammatory changes in the NC and FO groups. Dilated renal tubules, swollen tubular cells and vacuolar degeneration were seen in the SC and MO groups. Thickened basement membranes of renal tubules were also observed in these two groups (Figure 4B). The quantified renal injury score of the FO group was significantly lower than the SC and MO groups, but was not different from the NC group (Figure 4C).

## 4. Discussion

In this study, mice subjected to CLP-induced sepsis followed by fluid resuscitation and antibiotic treatment all survived the experiment, as witnessed at 72 h post-CLP. In a previous report, the same model without antibiotic treatment resulted in 75%–88% survival by 48 h after CLP [23]. It can be expected that the use of antibiotics improves survival. Since antibiotic therapy is the standard of care for critically-ill patients with polymicrobial sepsis, our experimental design may be more clinically suited for the study of sepsis-induced remote organ injury, which is usually late at onset and frequently deteriorates despite appropriate antibiotic treatment [24]. In this study, we administered fat emulsion via IP injection. Previous studies have demonstrated that fat emulsion given as a bolus IP injection could be absorbed by the peritoneal cavity and allows a high rate of absorption and bioavailability of the fat emulsion administered [25,26]. This study assessed the therapeutic use of fish oil-containing fat emulsion as a pharmaconutrient in addition to standard chow and water given *ad libitum*. The findings showed that treatment with fish oil-based emulsion produced some changes in the T lymphocyte subsets and modulated the expression of kidney’s inflammatory mediators, both of which were not observed in septic mice treated with saline.

T cells are important in the proper development of cellular and humoral immunity. Previous studies revealed that sepsis resulted in depletion of T lymphocyte with impaired T cell function [27], enhancement of Th1-Th17 expression to exaggerate inflammation [28] and an increase in Treg cells [29]. Our results are consistent with previous reports demonstrating that neutrophils were activated and Th1/Th17/Treg percentages were increased during sepsis, whereas blood T lymphocytes and CD8+ cells were decreased. However, when treated with fish oil-based fat emulsion, the Treg percentage remained persistently low, even by the late phase of sepsis. In addition, a reversal of the decrease in total T, CD4+ and CD8+ cells was observed. Treg cells are a distinct T cell subset with opposing properties to Th17. Tregs inhibit the effector response mediated by CD4+ and CD8+ cells. Considering the immunosuppressive properties, Tregs have an impact on the injury-induced immune dysfunction [30]. The lower Treg percentages and higher CD4+ in the FO group may indicate that the sepsis-induced immunosuppression was mitigated, and the CD4+ cell population recovered when fish oil emulsion was administered. A previous study also found that DHA diminished the suppressive capacity of Treg cells in a dose-dependent manner [31].

We analyzed the biochemical markers of renal and liver functions in this study. The elevated ALT and AST levels indicated that liver dysfunction occurred in the present experimental model. BUN is a conventional indicator for detecting renal dysfunction. It is considered a non-specific marker [32], and its levels did not rise significantly in the sepsis groups in this study. However, plasma Cr levels did rise, consistent with the elevation of NGAL. NGAL is a protein generally expressed in many tissues, including the kidney, lung and colon. Under normal conditions, NGAL expression is very low, but it increases greatly during epithelial injury and inflammation [33,34]. NGAL is considered a useful early biomarker for AKI, because it has been shown to predict the severity and progression of AKI in the acute care setting [34,35]. The findings of this study showed that plasma NGAL levels increased several fold after CLP, suggesting that renal injury occurred under the septic condition. Although NGAL levels were reduced in all septic groups by 72 h, its significant reduction in the FO-3 group compared to the SC-3 group is suggestive of attenuated inflammation following fish oil treatment.

In this study, upregulated expressions of HMGB1, TLR4, MyD88 and TNF-α mRNA were evident in renal tissues of the septic groups. HMGB1 is a nuclear protein that has been identified as a potent inflammatory mediator [36]. HMGB1 can bind to receptors, resulting in signal transduction that amplifies the inflammatory response. TLR4 is the critical receptor mediating the inflammatory activity of HMGB1 [37]. MyD88 is an adaptor protein for TLRs and acts as a link between the receptors and the downstream kinase. The HMGB1-mediated signal transduction is MyD88 dependent and leads to activation of NF-κB [38]. The low kidney injury score observed in the FO-3 group may be explained by the downregulated expression of the aforementioned renal inflammatory mediators along with enhanced HSP70 and PPAR-γ mRNA expressions. HSPs are highly conserved proteins involved in the mechanisms of cellular protection. A previous study found that increased serum levels of HSP70 correlated with improved survival in patients with severe trauma [39]. PPARs are nuclear receptors involved in lipid metabolism. *n*-3 PUFAs are shown to be ligands of PPAR-γ, which can activate PPAR [40]. Induction of PPAR-γ expression is correlated with inhibition of NF-κB activity and, thus, reduces downstream inflammatory mediator production [41]. It is possible that, under the condition of sublethal CLP plus antibiotic treatment, the inflammatory process was suppressed more effectively by the higher dosage of fish oil provided by Omegaven as compared to the mixed oil lipid emulsion, thus better attenuating remote renal damage. An *ex vivo* study showed that a four-hour pre-treatment with a fish oil-based emulsion markedly reduced the production of the endothelial inflammatory mediator and concomitantly increased HSP72 expression [42]. Fish oil infusion leads to a rapid incorporation of *n*-3 fatty acids in cell membrane phospholipids and displacement of *n*-6 fatty acids from the cell membranes of immune cells [43], which may be the major factors in modulating inflammatory reaction and T cell response.

The downregulated expression of inflammatory mediators observed in the FO group cannot be explained by immunosuppression. We observed that the FO group had higher inflammatory cytokine levels in PLF than the saline group after CLP. Because the site of injury is the abdomen, the local inflammatory reaction may differ from systemic and remote organ responses. The higher inflammatory cytokines secreted by immune cells activate the immune response, leading to bacteria elimination in the abdomen. This may consequently alleviate systemic and remote organ inflammation. In a CLP model, Craciun *et al.* [44] demonstrated that increasing local neutrophil recruitment through IP injection of chemokines significantly enhances peritoneal bacterial clearance and improves survival. It is speculated that fish oil may have a favorable immune modulating effect on strengthening the local inflammatory responses while attenuating systemic inflammation. A previous study found that the mixture of MCT/LCT/*n*-9/*n*-3 (SMOFlipid) seemed to be neutral and have no deleterious effects on immune cells [45]. In this study, we noticed that the mixed oil fat emulsion is capable of producing similar effects in the percentage distribution of leukocyte subtypes (increased Th1 and Th17 and decreased Treg cells), in the release of inflammatory mediators (increased IL-6) and in the expression of inflammation-related genes in kidney (attenuated TLR-4 and TNF-α, suppressed MyD88 and HMGB1, preserved PPAR-γ and induction of HSP70) comparable to those found in the FO group. However, there are also variables observed in the FO group that differ significantly from the SC group that were not found when the mixed oil fat emulsion was used. These findings may suggest that the utilization of mixed oil results in some influences on inflammatory and immune responses under the septic condition. The dosage of mixed oil used in this study was 1 g/kg BW, which is relatively considered as a low dose, and mixed oil contains only 15% fish oil among the total lipids, which may not be potent enough to exert substantial influences on blood T cell subsets and renal inflammation. Whether a higher dosage of mixed oil may exert favorable effects on sepsis-induced inflammation requires further investigation.

## 5. Conclusions

This study showed that sublethal CLP-induced sepsis resulted in the activation of pro-inflammatory responses in the renal parenchyma, leading to significant histopathological damage in the renal tubules. Daily IP bolus injection of a fish oil-based fat emulsion as a pharmaconutrient following CLP was associated with retained circulating total T and CD4+ cell populations, reversed elevated Treg expression, upregulated HSP70 and PPAR-γ mRNA, downregulated inflammatory mediator expressions and attenuated septic renal histology. These results suggest that therapeutic use of a fish oil-based fat emulsion may modulate the inflammatory responses and offer protection against acute renal injury under conditions of antibiotic-treated polymicrobial sepsis.

## Figures and Tables

**Table 1:** Fatty acid composition of the fish oil-based (FO) and mixed oil (MO) lipid emulsions (% of total FA).

Fatty Acids	FO (Omegaven^®^)	MO (SMOFlipid^®^)
C8:0		16.3
C10:0		11.4
C14:0	4.9	
C16:0	10.7	9.2
C18:0	2.4	2.7
C16:1 *n*-7	8.2	
C18:1 *n*-9	12.3	27.8
C18:2 *n*-6	3.7	18.7
C18:3 *n*-3	1.8	2.4
C20:4 *n*-6	2.6	0.5
C20:5 *n*-3 (EPA)	18.8	2.4
C22:5 *n*-3	2.8	
C22:6 *n*-3 (DHA)	16.5	2.2
Others	15.3	6.4

Notes: EPA, eicosapentaenoic acid; DHA, docosahexaenoic acid.

**Table 2:** Primer sequences used in the quantitative real-time reverse-transcription polymerase chain reaction assays.

Gene Name	Accession No.	5′-3′Primer Sequence
β-actin	NM_007393.2	F: AGCCATGTACGTAGCCATCC R: CTCTCAGCTGTGGTGGTGAA
HMGB1	NM_010439.2	F: TTGCTTTGCCCATTTTGGGTCACA R: CCACAATGGCAGGGCATGTGGA
HSP70	NM_010479.2	F: GCTGGCTAGGAGACAGATATGTGGC R: AAAGCCCACGTGCAATACACAAAGT
MyD88	NM_010851.2	F: CATGGTGGTGGTTGTTTCTGAC R: TGGAGACAGGCTGAGTGCAA
TLR4	NM_021297.2	F: AGAAATTCCTGCAGTGGGTCA R: TCTCTACAGGTGTTGCACATGTCA
PPAR-γ	NM_011146.3	F: GCCCTTTGGTGACTTTATGG R: CAGCAGGTTGTCTTGGATGT
TNF-α	NM_013693.2	F: CCCTCACACTCAGATCATCTTCT R: GCTACGACGTGGGCTACAG

Notes: HMGB1, high mobility group box protein-1; HSP70, heat shock protein 70; MyD88, myeloid differentiation factor 88; TLR4, toll-like receptor 4; PPAR-γ, peroxisome proliferator-activated receptor; TNF-α, tumor necrosis factor-α; F, forward primer; R, reverse primer.

**Table 3:** Plasma concentrations of biochemical markers.

	NC	SC-1	FO-1	MO-1	SC-3	FO-3	MO-3
BUN (mg/dL)	24.3 ± 0.98	19.2 ± 0.8	21.0 ± 1.2	20.2 ± 1.2	22.3 ± 1.7	20.5 ± 1.6	22.1 ± 1.5
Cr (mg/mL)	4.56 ± 1.33 *	6.01 ± 1.02	7.16 ± 1.22	7.22 ± 0.96	9.83 ± 0.91 ^†^	6.33 ± 0.95	6.57 ± 0.73
ALT (U/L)	53.3 ± 5.0 *	142.0 ± 7.7	157.4 ± 8.4	158.4 ± 10.0	71.5 ± 7.9	86.4 ± 5.8	92.2 ± 5.9
AST (U/L)	91.2 ± 11.1 *	419.8 ± 41.2	372.8 ± 20.9	401.1 ± 15.8	193.7 ± 16.8	213.0 ± 11.5	232.6 ± 16.1
NGAL (ng/mL)	0.17 ± 0.07 *	19.66 ± 1.61	22.07 ± 0.78	19.78 ± 1.28	8.56 ± 1.23	6.74 ± 0.84 ^‡^	7.58 ± 1.19

Notes: Data are expressed as the mean ± SEM. N = 8 for NC, and *n* = 10 for the other experimental sepsis groups. NC, normal control group; SC, septic group with saline injection; FO, septic group with fish oil (Omegaven) injection; MO, septic group with mix oil (SMOFlipid) injection. SC-1, FO-1, MO-1: sacrificed at 1 day after cecal ligation and puncture (CLP); SC-3, FO-3, MO-3: sacrificed at 3 days after CLP. * Significantly different from all of the sepsis groups (*p* < 0.05). ^†^ Significantly different from the other 2 groups at the same time point (*p* < 0.05). ^‡^ Significantly different from the SC-3 group (*p* < 0.05).

**Table 4:** Inflammatory mediator concentrations in plasma and peritoneal lavage fluid (PLF).

	NC	SC-1	FO-1	MO-1	SC-3	FO-3	MO-3
Plasma							
PGE_2_ (μg/mL)	2.17 ± 0.31	1.36 ± 0.15	0.95 ± 0.11	1.33 ± 0.16	1.45 ± 0.45	1.79 ± 0.67	1.49 ± 0.44
Lipoxin A_4_ (pg/mL)	ND	9.58 ± 3.95	8.52 ± 2.72	9.69 ± 4.80	ND	ND	ND
PLF (pg/mL)							
IL-1β	ND	48.0 ± 5.4	58.9 ± 7.4	53.0 ± 6.5	20.3 ± 4.5	55.2 ± 7.3 ^‡^	28.4 ± 7.8
TNF-α	ND	5.5 ± 0.5	6.0 ± 0.5	6.7 ± 1.6	5.3 ± 0.7	9.3 ± 2.0 ^‡^	6.2 ± 0.7
IL-6	ND	738.3 ± 70.6	1288.2 ± 72.8 ^†^	1052.8 ± 168.3 ^†^	185.8 ± 43.1	239.8 ± 54.7	275.2 ± 30.9
IL-10	91.8 ± 55.3 *	19.3 ± 3.5	40.0 ± 10.5 ^‡^	25.0 ± 5.5	6.5 ± 1.4	12.7 ± 2.6	11.5 ± 3.2

Notes: Data are expressed as the mean ± SEM. N = 8 for NC, and *n* = 10 for other experimental septic groups. NC, normal control group; SC, septic group with saline injection; FO, septic group with fish oil (Omegaven) injection; MO, septic group with mix oil (SMOFlipid) injection. SC-1, FO-1, MO-1: sacrificed at 1 day after CLP; SC-3, FO-3, MO-3: sacrificed at 3 days after CLP. PGE_2_: prostaglandin E_2_; TNF-α: tumor necrosis factor-α; IL: interleukin; ND: not detectable. * Significantly different from all of the sepsis groups (*p* < 0.05). ^†^ Significantly different from the SC-1 group (*p* < 0.05). ^‡^ Significantly different from the SC and MO groups at the same time point (*p* < 0.05).
